# Mass cytometry and single-cell RNA sequencing reveal immune cell characteristics of active and inactive phases of Crohn’s disease

**DOI:** 10.3389/fmed.2022.1064106

**Published:** 2023-01-12

**Authors:** Wenjia Lin, Shiying Liu, Zhuojian Huang, Haiwen Li, Tianyu Lu, Yongxin Luo, Jiamin Zhong, Zewen Xu, Yu Liu, Yanwu Li, Peiwu Li, Qian Xu, Jiazhong Cai, Huibiao Li, Xin-lin Chen

**Affiliations:** ^1^School of Basic Medical Sciences, Guangzhou University of Chinese Medicine, Guangzhou, China; ^2^Shenzhen Traditional Chinese Medicine Hospital, Shenzhen, China; ^3^Department of Medical Statistics, School of Public Health, Sun Yat-sen University, Guangzhou, China; ^4^Science and Technology Innovation Center, Guangzhou University of Chinese Medicine, Guangzhou, China; ^5^Pi-Wei Institute, Guangzhou University of Chinese Medicine, Guangzhou, China; ^6^The First Affiliated Hospital, Guangzhou University of Chinese Medicine, Guangzhou, China

**Keywords:** Crohn’s disease, CyTOF, scRNA-seq, immune cell, monocyte

## Abstract

**Objectives:**

For Crohn’s disease (CD), the alternation of the active phase and inactive phase may be related to humoral immunity and cellular immunity. This study aims to understand the characteristics of immune cells in patients with active CD (CDa) and inactive CD (CDin).

**Methods:**

Mass cytometry (CyTOF) and single-cell RNA sequencing (scRNA-seq) data about CDa, CDin, and healthy control (HC) were included. CyTOF analysis was performed to capture gated subsets, including T cells, T regulatory (Treg) cells, B cells, innate immune cells, and natural killer (NK) cells. Differential analysis was used to identify different immune cell subsets among CDa, CDin, and HC. ScRNA-seq analysis was used to verify the results of CyTOF. CD-related signaling pathways were obtained using KEGG pathway enrichment analysis. CellChat analysis was used to infer the cell communication network among immune cell subsets.

**Results:**

Compared to patients with CDin, patients with CDa had higher abundances of CD16^+^CD38^+^CD4^+^CXCR3^+^CCR6^+^ naive T cells, HLA-DR^+^CD38^+^IFNγ^+^TNF^+^ effector memory (EM) T cells, HLA-DR^+^IFNγ^+^ naive B cells, and CD14^++^CD11C^+^IFNγ^+^IL1B^+^ monocytes. KEGG analysis showed the similarity of pathway enrichment for the earlier four subsets, such as thermogenesis, oxidative phosphorylation, and metabolic pathways. The patients with CDin were characterized by an increased number of CD16^+^CD56^dim^CD44^+^HLA-DR^+^IL22^+^ NK cells. Compared to HC, patients with CDa demonstrated a low abundance of HLA-DR^+^CCR6^+^ NK cells and a high abundance of FOXP3^+^CD44^+^ EM Tregs. CellChat analysis revealed the interaction network of cell subsets amplifying in CDa compared with CDin.

**Conclusion:**

Some immune subsets cells were identified for CDa and CDin. These cells may be related to the occurrence and development of CD and may provide assistance in disease diagnosis and treatment.

## Introduction

Crohn’s disease (CD) is a type of inflammatory bowel disease (IBD) in which the lesion is mostly located in the terminal ileum and right colon, and its inflammation is typically segmental, asymmetrical, and transmural. Most patients exhibit symptoms such as chronic diarrhea, abdominal pain, fatigue, and weight loss initially. Half of them develop complications (including fibrotic strictures, enteric fistulae, and intestinal neoplasia) with time, which often affects patients’ quality of life and leads to surgery (1–3). With the rapid growth of population, urbanization, industrialization, and westernization of lifestyle, the CD became a global disease at the turn of the 21st century (4, 5). The rapid changes in CD epidemiology have placed a heavy burden on healthcare delivery worldwide (6).

The etiology of CD is unknown and may be related to impaired intestinal barrier function, dysregulated innate and adaptive immune responses, disturbances in the intestinal microbiota, and genetic factors (2). Many studies have explored the immune mechanism of CD. Some studies reported that T lymphocytes played a dominant role in the chronic inflammation process of CD (7, 8). Some studies pointed out that the imbalance of innate lymphoid cells (ILCs) in the gut led to CD (9, 10). However, immune regulation was a complex process, and immune cells did not exist independently but interacted with each other. Therefore, a multidimensional and in-depth explanation of the pathogenesis of CD will be of great significance.

Currently, the activity of CD is assessed based on standard clinical scores, which might not adequately reflect the immune mechanism of disease activity (11). To reveal the immunological characterization of the active phase and inactive phase for CD was momentous. However, few articles were performed to study immunological characteristics of the difference between CD patients with active phase (CDa) and those with inactive phase (CDin). Martin, Chang et al. conducted a study associated with resistance to anti-TNF therapy of CD by identifying an active cellular module (12), which inspired us that the determination of the immunological characteristics of CDa and CDin was of great significance for therapy.

Both mass cytometry (CyTOF) and single-cell RNA sequencing (scRNA-seq) were used to explore the immune characteristics of different stages of the disease. CyTOF is a high-throughput technique for measuring the abundance of proteins on the cell surface or within cells (13). CyTOF was applied to various functional assays, such as phenotypic characterization, intracellular cytokine determination, intracellular signaling state, and cell viability identification. Thus, the use of CyTOF offers new opportunities to address the complexity of *cellular* immunology (14). scRNA-seq is a new technology for amplification and sequencing the whole transcriptome at the single-cell level (15). Through scRNA-seq, various cell types can be acquired by learning the expression level and mutated structure of genes in individual cells. scRNA-seq plays a key role in the classification of cell types, identification of cellular immune microenvironment, and cell lineage evolution (16).

*Mitsialis et al.* performed a single-cell analysis of colon and blood samples from patients with IBD and identified different immune cell signatures of UC and CD, respectively (17). However, the immune characteristics of different stages of CD were unknown. This study intends to explore the immune cell characteristic of patients with CDa and CDin using CyTOF and scRNA-seq and to compare differences in immune cells among different disease periods of CD.

## Materials and methods

### CyTOF data analysis

#### Data preprocessing

Public data acquisition: Flow cytometry standard (FCS) files related to CD [including inactive CD (CDin) and active CD (CDa)] and healthy control (HC) samples were uploaded by the previous studies (17).

Flow cytometry standard files were downloaded and manually analyzed from Cytobank (18).^1^ The channel of each FCS file was scaled based on the appropriate proportion containing most of the signal in the identifiable area. Gated subsets were captured according to their corresponding recognizable positive or negative surface factors, including T cells (CD45^+^CD3^+^ cells), T regulatory (Treg) cells (CD45^+^CD8a^–^CD4^+^CD25^+^CD127^–^ cells), B cells (CD45^+^CD3^–^CD19^+^ cells), innate immune cells (CD45^+^CD3^–^CD19^–^ cells), and natural killer (NK) cells (CD45^+^CD3^–^CD56^+^ cells). The exported pretreated FCS files were uploaded to FlowJo software checking compensation and generated again for subsequent analysis.

#### Data analysis

The preprocessed data were analyzed using R software (version 4.1), including the following steps. (1) Data input: Processed FCS files were imported into R by the FlowCore package. FCS files with corresponding metadata were combined to build a SingleCellExperiment object using the CATALYST package (19). (2) Quality control: The consistency of the number of FCS files of antibodies was ensured. The signal value in diagnostic plots was ensured to be normalized, and multidimensional scaling (MDS) was performed to check the relationship among samples’ distance. (3) Clustering, dimension reduction, and visualization: Unsupervised cluster was used. Then, 1,000 cells from each sample were randomly selected for t-SNE dimensionality reduction, and the cells were colored according to clustering results. The heatmaps were drawn by ggplote2 for visualization, showing the expression of marker signals for different cell subsets in each cluster. (4) Statistical analysis and visualization: The cell abundance of each sample at each node based on different cell types was calculated. Kruskal–Wallis was used for the comparison of three groups, and the Wilcoxon rank-sum test was used for the comparison of two groups. The *p*-value of ≤ 0.05 was considered statistically significant. In visualization, the box plots representing sample proportion were used to depict the differences in expression among three groups (CDa, CDin, and HC).

### Single-cell RNA sequencing

#### Data preprocessing

Public data acquisition: Single-cell RNA data were obtained from two GEO databases. Six samples of CDa and CDin were obtained from GSE134809, and six HC samples were obtained from GSE152321. ScRNA-seq was conducted to verify the results of CyTOF.

Seurat v4.0 was employed to analyze single-cell RNA data.^2^ (1) Quality control: Sequence alignment and filtering of data were carried out in Seurat v4.0. CD45^+^ immune cells with nFeature_RNA more than 200, and at most 15% mitochondrial-specific genes, were retained. (2) Multiple data integration and standardization: Single-cell RNA data were normalized using SCTransform, and the highly variable genes for integration were selected. Anchor integration of two datasets by function IntegrateData was used to eliminate batch effect among the different datasets and samples, which resulted in a filtered data matrix of 3,000 genes and 23,072 cells for further analysis.

#### Verification of CyTOF results

We conducted a single-cell analysis to verify the statistically significant results of CyTOF. (1) Dimensionality reduction: Principal component analysis (PCA) and t-SNE were used for dimensionality reduction. The first 20 principal components were input for unsupervised clustering, and the t-SNE diagram was used for visualization. (2) Cell types annotation: Cell types were annotated according to the expression of commonly related markers in different clusters, which was also annotated using the SingleR built-in dataset MonacoimmuneData (20). (3) Statistical analysis: Cell subsets were grouped again according to the results of CyTOF; for instance, CD16^+^CD38^+^CD4^+^CXCR3^+^CCR6^+^ naive T cells were subsetted from T cells based on CyTOF results. The cell abundance of each sample at each node for different cell types was calculated. Then, Kruskal–Wallis and Wilcoxon rank-sum test analyses were used to verify whether the abundance difference of the three groups in the same subsets would agree with the CyTOF results.

#### KEGG analysis of DEGs

Compared with all other clusters, the Wilcoxon rank-sum test was used to identify differentially expressed genes (DEGs) in each cluster. Kyoto Encyclopedia of Genes and Genomes (KEGG) pathway enrichment analysis of DEGs was conducted using David^3^ and visualized by R package ggplot2.

#### CellChat analysis

In the previous research, four subsets increased significantly in patients with CDa compared to patients with CDin. In order to study the interaction between these four subsets and other cells under different phases, we carried out a cell communication analysis.

Seurat objects were built by using CD intestinal tissue samples from GEO134809. In cell type labeling, cell types were annotated, including T cells, B cells, NK cells, innate immune cells, epithelial cells, endothelial cells, and the four subsets with significant differences between CDa and CDin. CellChat package (21) was applied to convert the Seurat object into a CellChat object, infer the cell communication network among subsets, and visualize the single signal pathways of subset interaction.

## Results

CyTOF analysis was performed on the data of intestinal mucosa from 37 CD (CDin = 25 and CDa = 12) and 18 HC. The characteristics of demographic data were as follows. There were no significant differences between the three groups in factors such as gender, age at collection, age at diagnosis, and duration of disease among others (Supplementary Table 1).

Single-cell RNA sequencing data contained six HC and 12 patients with CD (CDin = 6 and CDa = 6). scRNA-seq was conducted to verify the results of CyTOF. Furthermore, CellChat analysis was applied to deduce the cell communication network among subsets by 22 CD samples (CDin = 11 and CDa = 11). There was no significant difference for factors of gender (Supplementary Table 1).

### CD16^+^CD38^+^CD4^+^ Naive T Cells, HLA-DR^+^CD38^+^IFNγ^+^TNF^+^ EM T Cells, and FOXP3^+^CD44^+^ EM tregs are enriched in CDa

**FIGURE 1:**
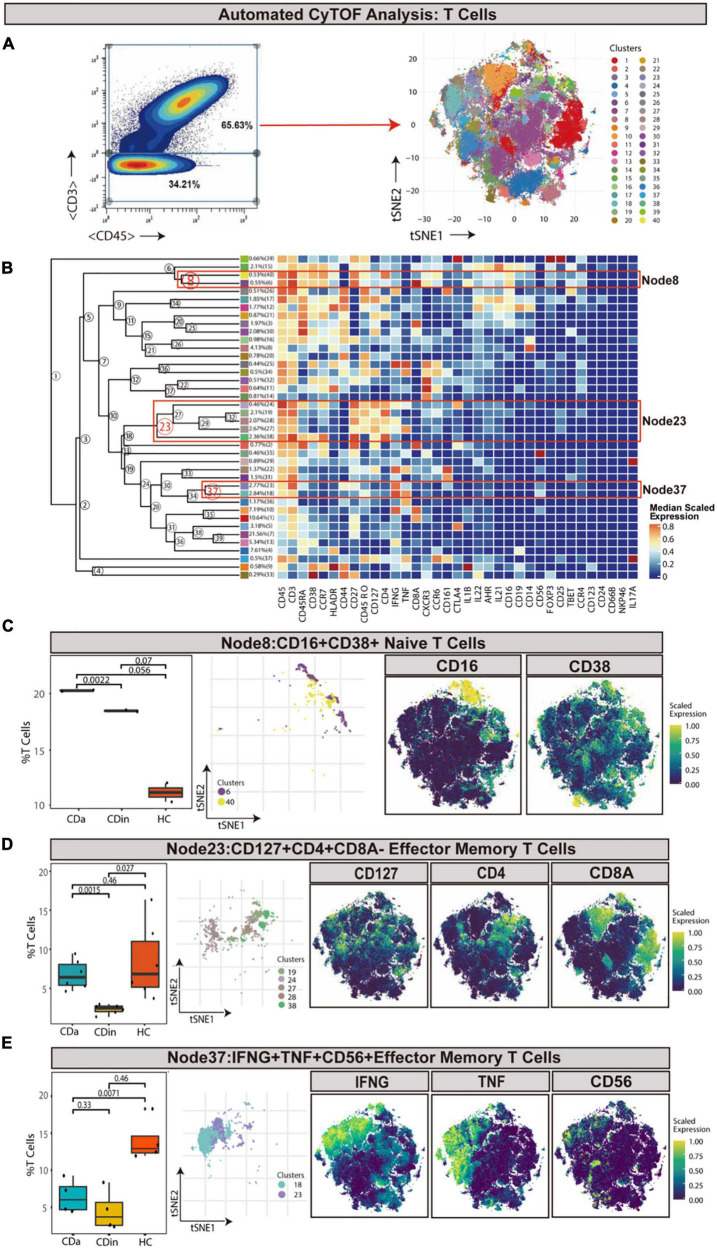
CyTOF analysis showed differences in T cells among CDa, CDin, and HC. **(A)** Gating strategy to determine T cells (left) and t-SNE projection of selected T cells derived from CyTOF data just for visualization purposes (right). **(B)** Cluster dendrogram and heatmap of selected T cells displaying median scaled expression levels of all markers in each cluster. Branch numbers correspond to the cluster numbers in the t-SNE plot [from panel **(A)**]. Selected nodes are circled in red, and the subset names are labeled on the right. **(C)** The percentage of selected Node8 in T cells among CDa, CDin, and HC groups with Wilcoxon rank-sum test results (left) and the t-SNE plot of Node8 with gene expression feature plot for CD16/CD38 (right). **(D)** The percentage of selected Node23 in T cells among three groups (left) and the t-SNE plot of Node23 with gene expression feature plot for CD127/CD4/CD8A (right). **(E)** The percentage of selected Node37 in T cells among three groups (left) and the t-SNE plot of Node37 with gene expression feature plot for IFNG/TNF/CD56 (right).

T cells were sorted out according to the manual gating of CD45^+^CD3^+^ (Figures 1A, B). CD16^+^CD38^+^CD4^+^ naive T cells (defined as CD45RA^+^CD45RP^–^CCR7^+^CD27^+^) were detected to synergistically overexpress CXCR3 and CCR6 cytokines, and they were enriched in CDa with statistical significance comparing with CDin (*P* < 0.05, Figure 1C). Node23 was a subset of effector memory (EM) T cells (defined as CD45RA^–^CD45RO^+^CCR7^–^CD27^±^), co-expressed CD127, IFNγ, and CD4 as well as a low-level of CD8. CD127^+^CD4^+^CD8A^–^ EM T cells were found to be significantly reduced in CDin than that in CDa and HC (*P* < 0.05, Figure 1D). However, there was no significant difference between CDa and HC. Node37 was a subset of EM T cells co-expressed IFNG, TNF, and CD56. IFNγ^+^TNF^+^CD56^+^ EM T cells were found significantly enriched in HC compared with both CDa and CDin (*P* < 0.05, Figure 1E), and there was no significant difference between CDa and HC.

**FIGURE 2:**
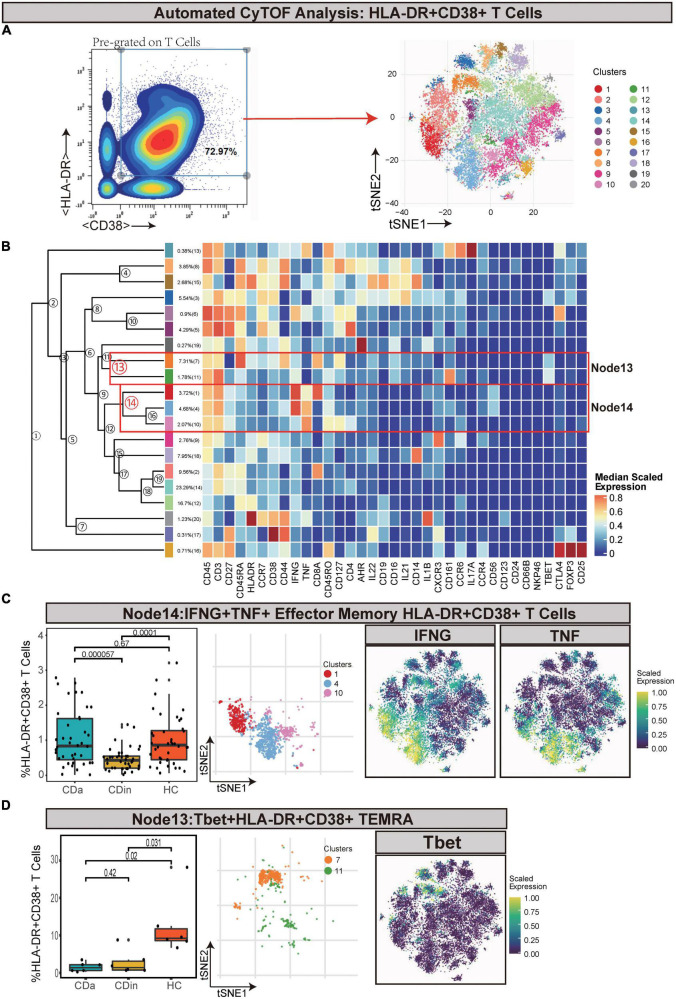
CyTOF analysis showed differences in HLA-DR + CD38 + T cells among CDa, CDin, and HC. **(A)** Gating strategy (left) and t-SNE projection (right) of HLA-DR + CD38 + T cells derived from CyTOF data. **(B)** Cluster dendrogram and heatmap of HLA-DR + CD38 + T cells. **(C)** The percentage of Node14 in HLA-DR + CD38 + T cells among three groups (left) and the t-SNE plot of Node14 with gene expression feature plot for IFNG/TNF (right). **(D)** The percentage of Node13 in HLA-DR + CD38 + T cells among three groups (left) and the t-SNE plot of Node13 with gene expression feature plot for Tbet (right).

Pre-gated on T cells, HLA-DR^+^CD38^+^ cells were ulteriorly gated and subdivided (Figures 2A, B). IFNγ^+^TNF^+^ EM T cells (Node14) were a subset of EM T cells that expressed IFNγ and TNF. IFNγ^+^TNF^+^ EM T cells demonstrated a lower abundance in CDin compared with CDa and HC (*P* < 0.05, Figure 2C). Node13 was a subset of HLA-DR^+^CD38^+^ effector memory cells re-expressing CD45RA (TEMRA), which also highly expressed Tbet. This subset was significantly higher in HC than both CDa and CDin (*P* < 0.05, Figure 2D).

**FIGURE 3:**
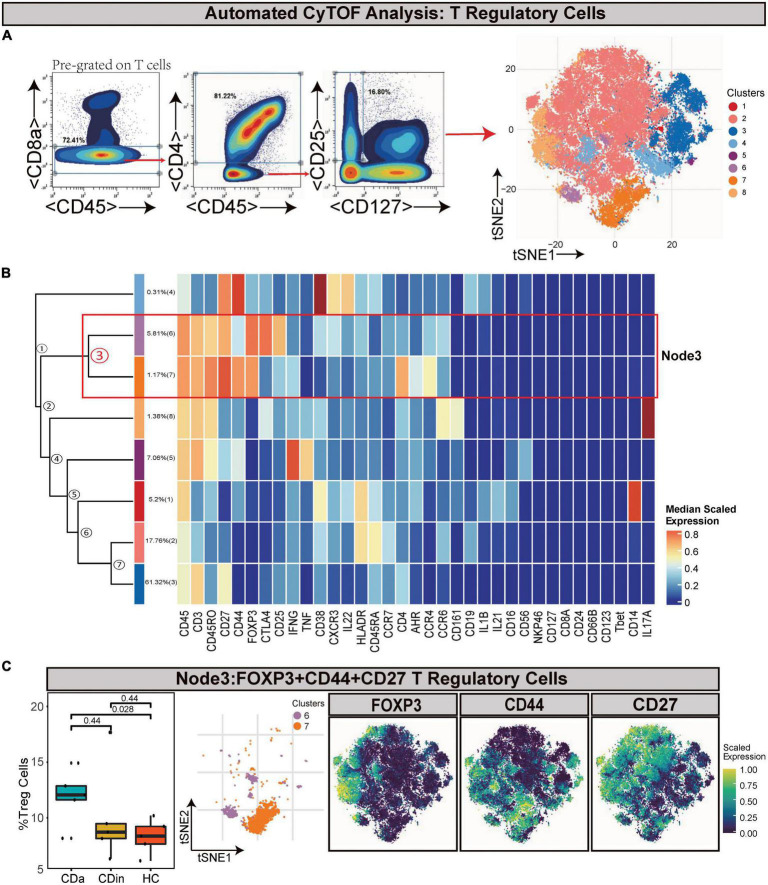
CyTOF analysis showed differences in T regulatory cells among CDa, CDin, and HC. **(A)** Gating strategy (left) and t-SNE projection (right) of T regulatory cells (Tregs) derived from CyTOF data. **(B)** Cluster dendrogram and heatmap of Tregs. **(C)** The percentage of Node3 in Tregs among three groups (left) and the t-SNE plot of Node3 with gene expression feature plot for FOXP3/CD44/CD27 (right).

Tregs (defined as CD45^+^CD8^–^CD4^+^CD25^+^CD127^–^) were manually gated based on T cells (Figures 3A, B). Node3 was FOXP3^+^CD44^+^ EM Tregs. FOXP3^+^CD44^+^ EM Tregs were observed to expand markedly in CDa compared with HC (*P* < 0.05, Figure 3C).

### HLA-DR^+^IFNγ^+^ naive B cells show a difference between CDa and CDin, while CXCR3^+^CCR6^+^HLA-DR^+^ naive B cells are less abundant in CDa

**FIGURE 4:**
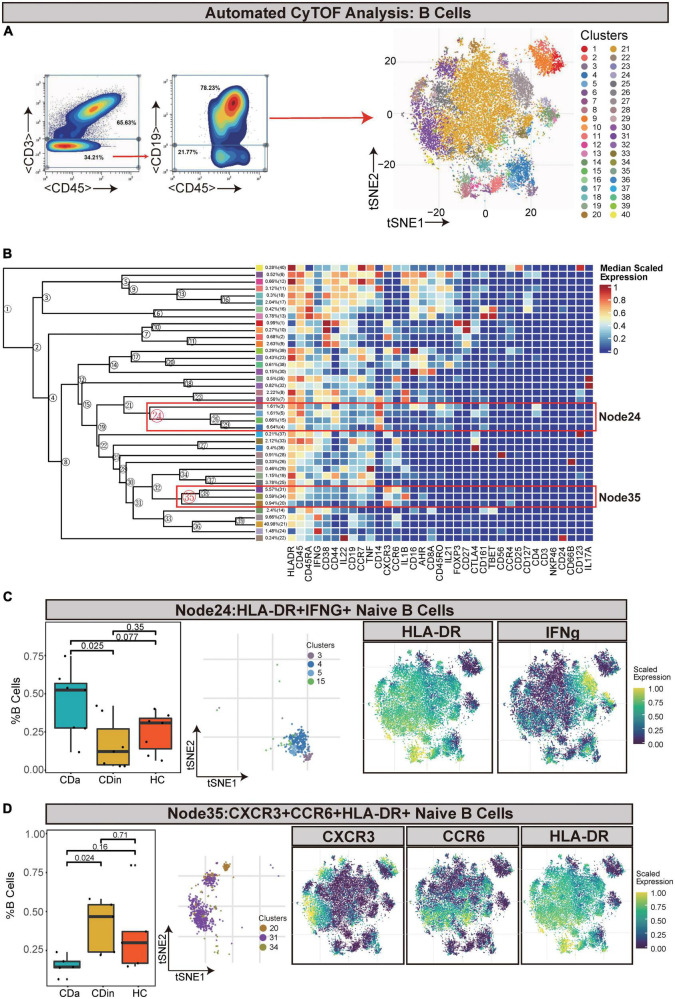
CyTOF analysis showed differences in naïve B cells among CDa, CDin, and HC. **(A)** Gating strategy (left) and t-SNE projection (right) of B cells derived from CyTOF data. **(B)** Cluster dendrogram and heatmap of B cells. **(C)** The percentage of Node24 in B cells among three groups (left) and the t-SNE plot of Node24 with gene expression feature plot for HLA-DR/IFNg (right). **(D)** The percentage of Node35 in B cells among three groups (left) and the t-SNE plot of Node35 with gene expression feature plot for CXCR6/CCR6/HLA-DR (right).

For the sake of delving more comprehensively into the discrepancy between active and inactive immune landscapes, B cells (defined as CD45^+^CD3^–^CD19^+^) were deliberately gated and classified for subsequent analysis (Figures 4A, B). Through comparison, B cell subsets with significant differences among three groups were obtained: HLA-DR^+^IFNγ^+^ naive B cells (Node24) and CXCR3^+^CCR6^+^HLA-DR^+^ naive B cells (Node35). HLA-DR^+^IFNγ^+^ naive B cells decreased in CDin compared with CDa (*P* < 0.05, Figure 4C). Although HLA-DR^+^IFNγ^+^ naive B cells tended to increase in CDa, there was no significant difference between CDa and HC. CXCR3^+^CCR6^+^HLA-DR^+^ naive B-cell subsets were more abundant in CDin than CDa (*P* < 0.05, Figure 4D).

### CDa is characterized by a copiousness of CD14^++^CD11C^+^IFNγ^+^ monocytes

**FIGURE 5:**
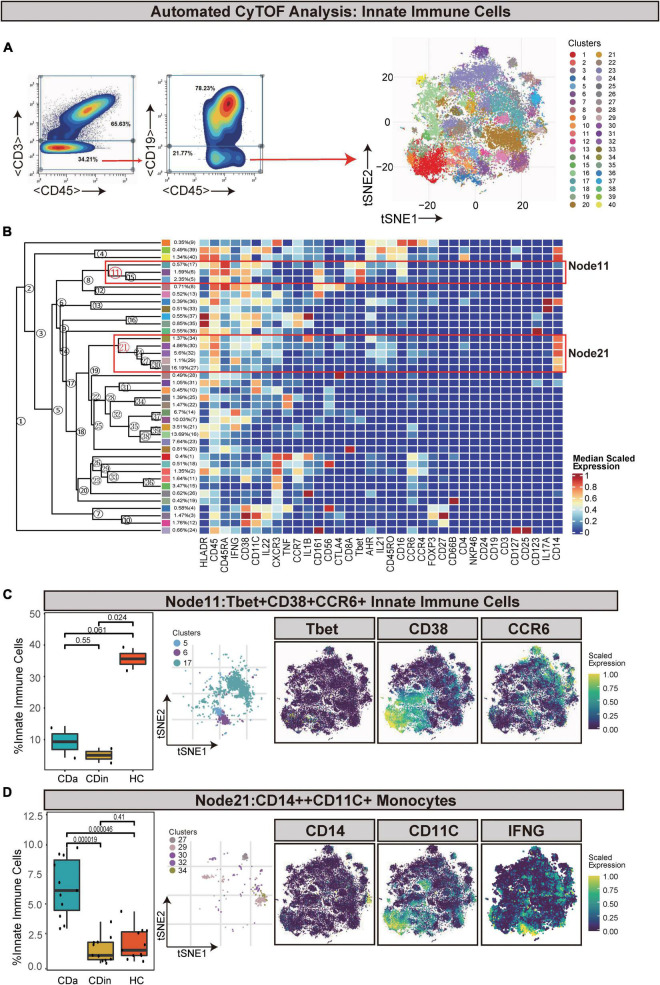
CyTOF analysis showed differences in immune cells among CDa, CDin, and HC. **(A)** Gating strategy (left) and t-SNE projection (right) of innate immune cells derived from CyTOF data. **(B)** Cluster dendrogram and heatmap of innate immune cells. **(C)** The percentage of Node11 in innate immune cells among three groups (left) and the t-SNE plot of Node11 with gene expression feature plot for Tbet/CD38/CCR6 (right). **(D)** The percentage of Node21 in innate immune cells among three groups (left) and the t-SNE plot of Node21 with gene expression feature plot for CD14/CD11c (right).

Innate immune cells (defined as CD45^+^CD3^–^CD19^–^) were manually gated and sorted out for a deep investigation (Figures 5A, B). Tbet^+^CD38^+^CCR6^+^ innate immune cells (Node11) were dramatically elevated in HC in comparison with CDa and CDin (*P* < 0.05, Figure 5C). In addition, IFNγ^+^IL1B^+^ monocytes (defined as CD14^++^CD11C^+^ innate immune cells) were labeled as Node21, and it was significantly high in CDa compared with CDin and HC (*P* < 0.05, Figure 5D).

### CD16^+^CD56^dim^ NK cells and HLA-DR^+^CCR6^+^ NK cells are diminished in CD

**FIGURE 6:**
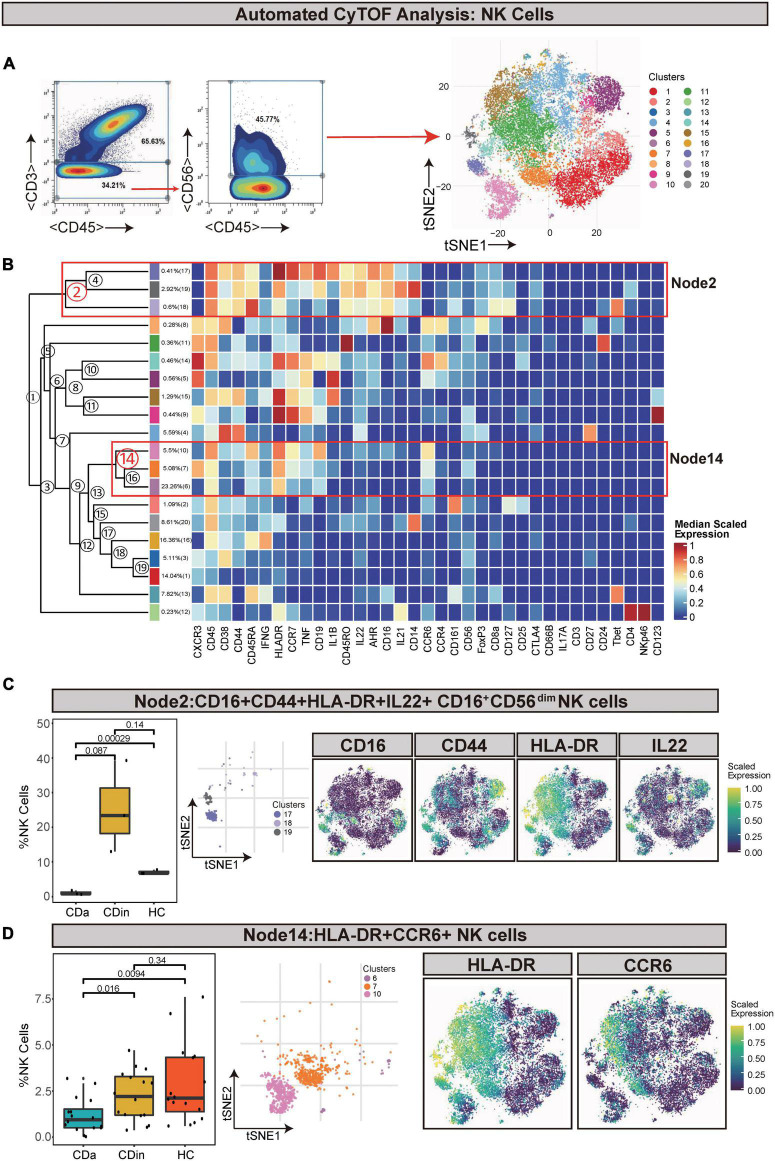
CyTOF analysis showed differences in NK cells among CDa, CDin, and HC. **(A)** Gating strategy (left) and t-SNE projection (right) of NK cells derived from CyTOF data. **(B)** Cluster dendrogram and heatmap of NK cells. **(C)** The percentage of Node2 in NK cells among three groups (left) and the t-SNE plot of Node2 with gene expression feature plot for CD16/CD44/HLA-DR/IL22 (right). **(D)** The percentage of Node14 in NK cells among three groups (left) and the t-SNE plot of Node14 with gene expression feature plot for HLA-DR/CCR6 (right).

Natural killer cells (defined as CD45^+^CD3^–^CD56^+^) were artificially captured according to the corresponding recognizable expression of cell surface factors (Figures 6A, B). CD16^+^CD56^dim^ NK cells (Node2) were a subset of NK cells and characterized by a high expression of CD16 and a low expression of CD56. CD16^+^CD56^dim^ NK cells also had CD44, HLA-DR, and IL22 co-expression. This subset presented a lower proportion in CDa than in CDin and HC (*P* < 0.05, Figure 6C), but there was no statistically significant difference between CDin and HC. HLA-DR^+^CCR6^+^ NK cells (Node14) also significantly diminished in CDa compared with CDin and HC (*P* < 0.05, Figure 6D).

### Single-cell RNA sequencing analysis verifies the difference in specific immune subsets in CyTOF results

**FIGURE 7:**
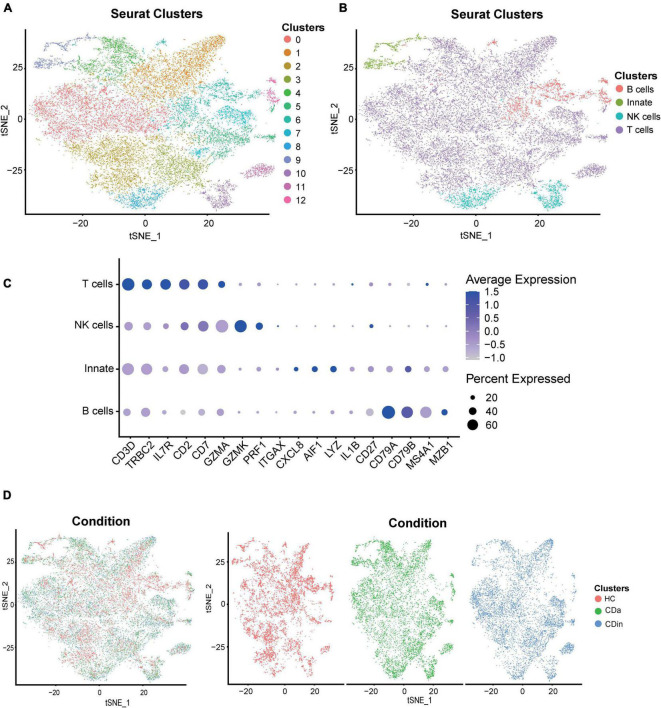
Recognition of diverse cells among CDa, CDin, and HC using scRNA-seq. **(A)** Visualized t-SNE plot derived from the clustering of CD and HC data in GSE13480 and GSE152321, colored and numbered depending on the clustering results. **(B)** The t-SNE plot is colored and labeled based on inferred cell type identity. **(C)** Dot plot depicting the expression levels of relevant genes in different cell clusters. **(D)** t-SNE plots show the distribution of tissue cells from different states (CDa, CDin, and HC).

For a more detailed investigation of diverse distribution patterns of immune cell subsets among CDa, CDin, and HC, we carried out a relevant single-cell analysis. Based on the retained CD45^+^ immune cells, four subsets were annotated according to the conventional immune-associated surface antigens, including T cells, B cells, NK cells, and innate immune cells (Figures 7A–C). The expression density of cell subsets among different groups (CDa, CDin, and HC) was observed (Figure 7D).

**FIGURE 8:**
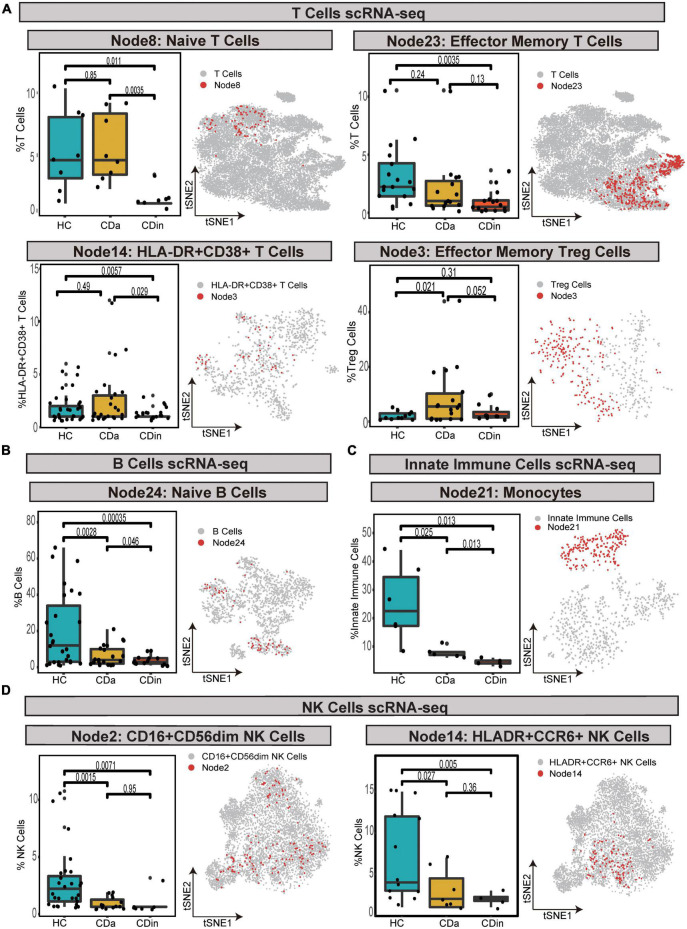
Validation of cell subsets differences between CD and HC by scRNA-seq. **(A–D)** Box plots of T-cell subsets **(A)**, B-cell subset **(B)**, an innate immune cells subset **(C)**, and NK cell subsets **(D)** abundances among CDa, CDin, and HC are shown on the left, with Wilcoxon rank-sum test results. t-SNE plots depicting the distribution of various cell subsets are shown on the right, respectively. The “Node” labels in the box plot and the t-SNE plot correspond to the CyTOF results.

For T cells, CD16^+^CD38^+^CD4^+^ naive T cells were confirmed to expand in CDa, and the abundance between CDa and CDin was conspicuous. CD127^+^CD4^+^CD8A^–^ EM T cells showed a decrease in CDin, with a significant difference compared with HC. HLA-DR^+^CD38^+^IFNγ^+^TNF^+^ EM T cells were enriched in CDa, which could be used to distinguish between CDa and CDin. FOXP3^+^CD44^+^ EM Tregs were significantly increased in CDa compared with HC (*P* < 0.05, Figure 8A).

For B cells, HLA-DR^+^IFNγ^+^ naive B cells had the highest abundance in HC and the lowest abundance in CDin. The statistical significance between CDa and CDin was verified (*P* < 0.05, Figure 8B).

For innate immune cells, CD14^++^CD11C^+^IFNγ^+^IL1B^+^ monocytes demonstrated a significantly notable increment in HC (*P* < 0.05). CD14^++^CD11C^+^IFNγ^+^IL1B^+^ monocytes dramatically increased in CDa compared with HC (*P* < 0.05, Figure 8C).

For NK cells, CD16^+^CD44^+^HLA-DR^+^IL22^+^ NK cells and HLA-DR^+^CCR6^+^ NK cells were both dramatically diminished in CDa compared to HC (*P* < 0.05, Figure 8D).

### KEGG analysis of differential genes has been carried out to investigate the functional expression of specific nodes in CD

The following subsets, which expanded substantially in CDa than CDin, were selected for KEGG analysis: CD16^+^CD38^+^CD4^+^CXCR3^+^CCR6^+^ naive T cells, HLA-DR^+^CD38^+^IFNγ^+^TNF^+^ EM T cells, HLA-DR^+^IFNγ^+^ naive B cells, and CD14^++^CD11C^+^IFNγ^+^IL1B^+^ monocytes.

**FIGURE 9:**
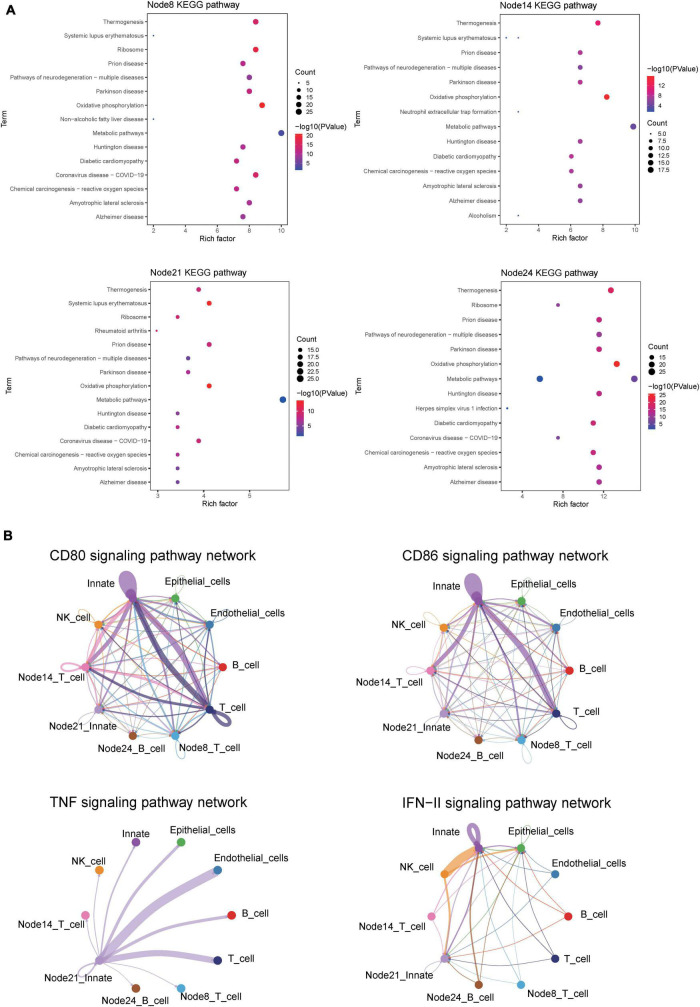
CellChat and KEGG revealed critical pathways that influence the course of CD. **(A)** KEGG pathway enrichment analysis of four cell subsets of interest. Node8:CD16 + CD38 + CD4 + CXCR3 + CCR6 + naive T cells; Node14: HLA-DR + CD38 + IFNγ + TNF + EM T cells; Node21:CD14 + + CD11C + IFNγ + monocyte; HLA-DR + IFNγ + naive B cells. **(B)** Circle plot showing the inferred CD80, CD86, IFNγ, and TNF signaling communication network among different cell subsets.

Kyoto Encyclopedia of Genes and Genomes analysis showed the similarity of pathway enrichment for the four subsets, such as thermogenesis, oxidative phosphorylation, and metabolic pathways (Figure 9A). Increased thermogenesis was a common feature of acute phase response, which was observed in injury, inflammation, infection, physical or emotional stress, and so on, and it corresponded to the active phase of CD. In addition, the oxidative phosphorylation system of mitochondria was rthe indispensable site of acute inflammation (22).

### CellChat analysis reveals the interaction network of subsets amplifying

The CellChat analysis was applied to speculate the interaction network of 10 subsets, including T cells, B cells, NK cells, innate immune cells, epithelial cells, endothelial cells, CD16^+^CD38^+^CD4^+^CXCR3^+^CCR6^+^ naive T cells, HLA-DR^+^CD38^+^IFNγ^+^TNF^+^ EM T cells, HLA-DR^+^IFNγ^+^ naive B cells, and CD14^++^CD11C^+^IFNγ^+^IL1B^+^ monocytes. In the overall performance of all selected cell groups, the number and weight/strength of cell interaction were statistically analyzed, and the signals sent by each cell subset were examined.

For CDa, the cell interaction network mediated was visualized using a single signal pathway, including CD80, CD86, IFNγ, and TNF signal pathway, which showed differences in built-in enrichment analysis or case–control (Figure 9B). For CDin, there is no significant communication of the TNF pathway.

## Discussion

Subsets with significant abundance differences among CDa, CDin, and HC were identified using CyTOF and scRNA-seq. The high abundance of CD16^+^CD38^+^CD4^+^CXCR3^+^CCR6^+^ naive T cells, HLA-DR^+^CD38^+^IFNγ^+^TNF^+^ EM T cells, HLA-DR^+^IFNγ^+^ naive B cells, and CD14^++^CD11C^+^IFNγ^+^IL1B^+^ monocytes were considered to be cellular immune characteristics for the patients with CDa. In addition, the expansion of CD16^+^CD56^dim^CD44^+^HLA-DR^+^IL22^+^ NK cells was considered to be a special subset for CDin.

The high abundance of CD16^+^CD38^+^CD4^+^CXCR3^+^ CCR6^+^ naive T cells was first found to be associated with CDa. According to our result, CD16^+^CD38^+^CD4^+^CXCR3^+^CCR6^+^ naive T cells were obviously increased in the patients with CDa compared with HC and CDin. The expansion of CD16^+^CD38^+^CD4^+^CXCR3^+^CCR6^+^ naive T cells might be related to the formation of T-helper 1 (Th1), which was relevant to the pathogenesis of CD. In IBD, naive T cells differentiate into various T-helper (Th) cells mediated by relevant antigen-presenting cells (23). Furthermore, an increase in CD16, CXCR3, and CCR6 was associated with inflammation in CDa. CD16 is a low-affinity Fc receptor that mediates phagocytosis and cytotoxicity and is a pharmacogenetic biomarker of anti-TNF therapy (24). CXCR3 was a chemokine expression receptor that determined the different functional states of CD4^+^ T cells and gave impetus to the formation of Th1 (25). In addition, CXCR3 could activate chemokines, such as CXCL9, CXCL10, and CXCL11, to recruit immune cells at the site of inflammation, causing local inflammation amplification and thus inducing a deterioration of clinical manifestations (26).

HLA-DR^+^CD38^+^IFNγ^+^TNF^+^ EM T cells may be a subset associated with immune dysregulation in CDa. This subset had an increment in the patients with CDa than those with CDin. The increase of activated EM T cells in CD realized multiple pro-inflammatory functions by the release of more cytokines, which might further enhance and perpetuate inflammation (27–29). Furthermore, activated EM T cells were promoted to secrete HLA-DR, CD38, TNF, IFNγ, and other cytokines, and these cytokines were strongly associated with further enhancement of the active phase of IBD (30). In addition, the results of CellChat showed that this subset was regulated as a receptor in the CD80/CD86 signaling pathway, which was inextricably linked to the activation of T cells (31, 32). The increase of EM T cells in CDa made the activation of effector cells increase, leading to immune dysregulation and persistent inflammation (27, 29). Therefore, HLA-DR^+^CD38^+^IFNγ^+^TNF^+^ EM T cells may be cellular immune characteristics for CDa.

CD14^++^CD11C^+^IFNγ^+^IL1B^+^ monocytes may be an influential subset affecting the transition between active and inactive phases of CD. Our results pointed out that CD14^++^CD11C^+^IFNγ^+^IL1B^+^ monocytes enriched in the patients with CDa. Coincidentally, Zhang et al. applied scRNA-seq and CyTOF to define unique cell populations in rheumatoid arthritis (RA) and found that monocytes with high expression of IFNγ and IL1B were significantly enriched in RA (33). (1) This might be the result of pro-inflammatory signals from the site of infection (including pro-inflammatory cytokines and microbial molecules), inducing monocytes migration, such as the high expression of adhesion molecules in the endothelium, allowing monocytes to migrate across the endothelium when inflammation occurs (34–36). It could be guessed that under pathological conditions, monocytes acquired inflammatory effector functions as a result of diverse combinations of cytokines in the microenvironment. (2) Moreover, CellChat results showed that CD14^++^CD11C^+^IFNγ^+^IL1B^+^ monocytes were a paramount signal transmitter in TNF signaling pathway, and its major receptors were endothelial cells and T cells, which corresponded with preceding findings (37). Sampaio EP et al. also mentioned that the interaction between T cells and monocytes enhanced TNF production, which might contribute to the secretion of pro-inflammatory cytokines in disease states (38). In addition, T cell–monocyte interactions modulated epithelial dysfunction (39).

CD16^+^CD56^dim^CD44^+^HLA-DR^+^IL22^+^ NK cells were considered to be a special subset for CDin. CyTOF results showed that CD16^+^CD56^dim^CD44^+^HLA-DR^+^IL22^+^ NK cells were markedly increased in the patients with CDin compared with CDa, which was not mentioned before. Berahovich et al. reported that CD16^+^CD56^dim^ NK cells had strong toxicity (40), which might be more toxic when combined with the high expression of CD44 (41, 42). Previous studies reported that the killing capacity of peripheral circulating NK cells in patients with IBD was abated, which might lead to an increased risk of secondary infection for patients with IBD (43). In addition, the high expression of CD44 indicated that this subset had strong cytotoxicity (41, 42). The high expression of IL22 protected the integrity of the intestinal mucosal barrier (44, 45).

Our study pointed out that IFNγ played a momentous role in the pathogenesis of CD. IFNγ was highly expressed in four subsets (CD16^+^CD38^+^CD4^+^CXCR3^+^CCR6^+^ naive T cells, HLA-DR^+^CD38^+^IFNγ^+^TNF^+^ EM T cells, HLA-DR^+^IFNγ^+^ naive B cells, and CD14^++^CD11C^+^IFNγ^+^IL1B^+^ monocytes). Moreover, the four subsets were notably expanded in CDa compared with CDin. The results were confirmed by other research (46). Studies showed that IFNγ could enhance innate immune cell activation and recruitment to infected tissues. It could directly or indirectly induce the secretion of pro-inflammatory cytokines, antigen presentation, effector molecule expression, and the differentiation of each innate cell subpopulation (47). The high expression of IFNγ might keep pace with the increase of these cell subsets in the active phase of CD.

Although there are some interesting discoveries in the study, further mechanistic studies or experimental validation are essential to identify the pathogenic factors or biomarkers associated with a different phase of CD.

In conclusion, the high abundance of CD16^+^CD38^+^CD4^+^CXCR3^+^CCR6^+^ naive T cells, HLA-DR^+^CD38^+^IFNγ^+^TNF^+^ EM T cells, HLA-DR^+^IFNγ^+^ naive B cells, and CD14^++^CD11C^+^IFNγ^+^IL1B^+^ monocytes were considered to be cellular immune characteristics for CDa. The expansion of CD16^+^CD56^dim^CD44^+^HLA-DR^+^IL22^+^ NK cells was considered to be a special subset for CDin. These cells may be related to the changes in the active and inactive phases of CD. We hope that the results can provide assistance in disease diagnosis and treatment for patients with CD in the future.

## Data availability statement

The original contributions presented in this study are included in the article/Supplementary material, further inquiries can be directed to the corresponding author.

## Author contributions

X-LC and WL were responsible for the conception and design of the study. WL was responsible for the data processing and analysis. X-LC, WL, SL, and JZ wrote the manuscript. TL and ZX modified the figures. X-LC, WL, HWL, ZH, YXL, YL, YWL, PL, QX, and HBL had provided professional suggestions. All authors contributed to the writing of this manuscript and approved the final version of the manuscript.

## Footnotes

1
https://www.cytobank.org/

2
https://satijalab.org/seurat/

3
https://david.ncifcrf.gov/
