# Supplementary material for: Mass cytometry and single-cell RNA sequencing reveal immune cell characteristics of active and inactive phases of Crohn’s disease
Source: Front Med (Lausanne). 2023 Jan 12;9:1064106. doi: 10.3389/fmed.2022.1064106 (PMC9878392; doi:10.3389/fmed.2022.1064106)
Supplement: Supplementary file 1 [file Table_1.pdf]

Supplementary Table 1. The demographic characteristics of CyTOF data ,GSE152321 and GSE134809.

	**CDa**	**CDin**	**HC**
**CyTOF**			
**Number of samples**	12	35	18
**Gender** (n%)			
Female	5 (42%)	9 (36%)	12 (67%)
Male	7 (58%)	16 (64%)	6 (33%)
**Age at collection;** years (mean ±SD)	(20.6 ± 15.2)	(18.4 ± 6.6)	(24.6 ±17.3)
**Duration of disease;** years (mean ±SD)	(6.3 ± 10.9)	(5.1 ± 4.7)	(N/A)
**Histological disease activity**(Nancy Index), n (%)			
Nancy Index 0	0	20 (80%)	N/A
Nancy Index 1	0	5 (20%)	N/A
Nancy Index 2	8 (66%)	0	N/A
Nancy Index 3	2 (17%)	0	N/A
Nancy Index 4	2 (17%)	0	N/A
Unavailable	0	0	N/A
**Sample location** n (%)			
Left Colon	1 (8%)	2 (8%)	3 (17%)
Transverse Colon	11 (92%)	23 (92%)	15 (83%)
**GSE152321**			
**Number of samples**	0	0	6
**Gender** (n%)			
Female	N/A	N/A	3(50%)
Male	N/A	N/A	3 (50%)
**Age**: years (mean ±SD)	N/A	N/A	(46.2 ±13.0)
**GSE134809**			
**Number of samples**	6	6	0
**Gender** (n%)			
Female	2 (33%)	2 (33%)	N/A
Male	4 (67%)	4 (67%)	N/A
**Age**: years (mean ±SD)	(32.7 ± 12.9)	(32.7 ± 12.9)	N/A
